# The deubiquitinating enzyme USP25 binds tankyrase and regulates trafficking of the facilitative glucose transporter GLUT4 in adipocytes

**DOI:** 10.1038/s41598-019-40596-5

**Published:** 2019-03-18

**Authors:** Jessica B. A. Sadler, Christopher A. Lamb, Cassie R. Welburn, Iain S. Adamson, Dimitrios Kioumourtzoglou, Nai-Wen Chi, Gwyn W. Gould, Nia J. Bryant

**Affiliations:** 1 Department of Biology, University of York, York, YO10 4HJ UK; 2 Henry Wellcome Laboratory of Cell Biology, Institute of Molecular Cell and Systems Biology, College of Medical Veterinary and Life Sciences, University of Glasgow, Glasgow, G12 8QQ UK; 3 Department of Medicine, University of California, San Diego, La Jolla, CA 92093 USA

## Abstract

Key to whole body glucose homeostasis is the ability of fat and muscle cells to sequester the facilitative glucose transporter GLUT4 in an intracellular compartment from where it can be mobilized in response to insulin. We have previously demonstrated that this process requires ubiquitination of GLUT4 while numerous other studies have identified several molecules that are also required, including the insulin-responsive aminopeptidase IRAP and its binding partner, the scaffolding protein tankyrase. In addition to binding IRAP, Tankyrase has also been shown to bind the deubiquinating enzyme USP25. Here we demonstrate that USP25 and Tankyrase interact, and colocalise with GLUT4 in insulin-sensitive cells. Furthermore depletion of USP25 from adipocytes reduces cellular levels of GLUT4 and concomitantly blunts the ability of insulin to stimulate glucose transport. Collectively, these data support our model that sorting of GLUT4 into its insulin-sensitive store involves a cycle of ubiquitination and subsequent deubiquitination.

## Introduction

The facilitative glucose transporter GLUT4 expressed in fat and muscle is responsible for the increased rate of glucose transport into these cells in response to insulin^1^. In the absence of insulin, GLUT4 is retained intracellularly through active sequestration away from the general endosomal system into specialised GLUT4 storage vesicles (GSVs). It is from GSVs that GLUT4 translocates to the cell surface upon insulin binding its receptor. Following attenuation of the insulin signal, GLUT4 is again sequestered internally until a further insulin signal is received. This regulated trafficking underpins insulin-dependent postprandial clearance of plasma glucose and is defective in the disease states of insulin-resistance and Type-2 diabetes^2^.

GLUT4 enters GSVs from the *trans* Golgi network (TGN) in a trafficking step requiring the GGA (Golgi-localised γ-ear-containing, ARF-binding) family of clathrin adaptors^3,4^. We have previously reported that ubiquitination of GLUT4 is also required for its sorting into GSVs; with a ubiquitin-resistant version of GLUT4 failing to enter GSVs and consequently not exhibiting insulin-stimulated translocation to the cell surface^5^. Given that GGAs facilitate delivery of cargo proteins from the TGN into the endosomal system through recognition of attached ubiquitin moieties^6,7^, we proposed that ubiquitinated GLUT4 is sorted into the endosomal system by GGA proteins and that this is a critical step for sorting into GSVs^5^. A caveat with this model is that ubiquitination serves as a signal for lysosomal delivery of proteins *via* the multivesicular body (MVB) pathway^7^; this poses the question as to why ubiquitination doesn’t target GLUT4 for lysosomal and degradation.

Ubiquitination is a reversible modification, and in the same way that kinases and phosphatases allow for transient phosphorylation of proteins, opposing action of ubiquitin ligases and deubiquininases (DUBs) can regulate ubiquitination of specific substrates^8^. Coordinated activity of ubiquitin ligases and DUBs allows cells to fine tune down regulation of certain surface receptors by targeting ubiquitinated receptors to the lysosome via the MVB pathway while recycling a deubiquitinated population back to the cell surface^8^. Despite ubiquitination being absolutely required for sorting of GLUT4 into GSVs in adipocytes, only ~0.1% of the transporter is ubiquitinated at steady state^5^; an observation consistent with ubiquitination of GLUT4 being a transient modification. In this study we test a new hypothesis that once ubiquitination has served to direct GLUT4 into GSVs, subsequent deubiquitination prevents lysosomal degradation of the transporter.

To identify a candidate DUB for this process we turned to studies addressing the role of the insulin-responsive aminopeptidase IRAP in GLUT4 traffic. Like GLUT4, IRAP localises to GSVs and translocates to the plasma membrane in response to insulin^9^. Depletion of IRAP using siRNA in 3T3-L1 adipocytes results in reduced insulin-stimulated translocation of GLUT4^10,11^ and adipose tissue of IRAP knockout mice has less GLUT4 in the GSV-enriched low-density microsomal fraction than wild type control^12^, implicating IRAP in GLUT4 sorting. IRAP binds to the scaffolding protein tankyrase (TNKS)^13^ whose knockdown also abrogates insulin-stimulated GLUT4 delivery to the cell surface of adipocytes^11^. Binding partners of TNKS, including IRAP, contain a conserved TNKS binding motif (RXX(P/A)DG)^14^. Intriguingly, this motif resides in the C-terminus of USP25^14^, a deubiquitinase that has been identified as a TNKS binding protein in HEK293 cells^15^. Binding of both USP25 and IRAP to TNKS in adipocytes could serve to recruit USP25 to GSVs. This is an attractive model given that IRAP is required for the formation of GSVs^10^. USP25, thus positioned, could then serve to deubiquitinate GLUT4, rescuing the transporter from lysosomal degradation.

Here we have used insulin-sensitive 3T3-L1 adipocytes to test this hypothesis. Consistent with our model, we demonstrate a direct interaction between USP25 and TNKS in adipocytes, both of which cofractionate with a subpopulation of internal GLUT4-containing membranes. We also demonstrate that USP25 is required for insulin-stimulated glucose transport into 3T3-L1 adipocytes and that knockdown of USP25 results in reduced cellular levels of GLUT4 protein.

## Results

### The RXXPDG motif within USP25 is required for TNKS binding in adipocytes

**Figure 1:**
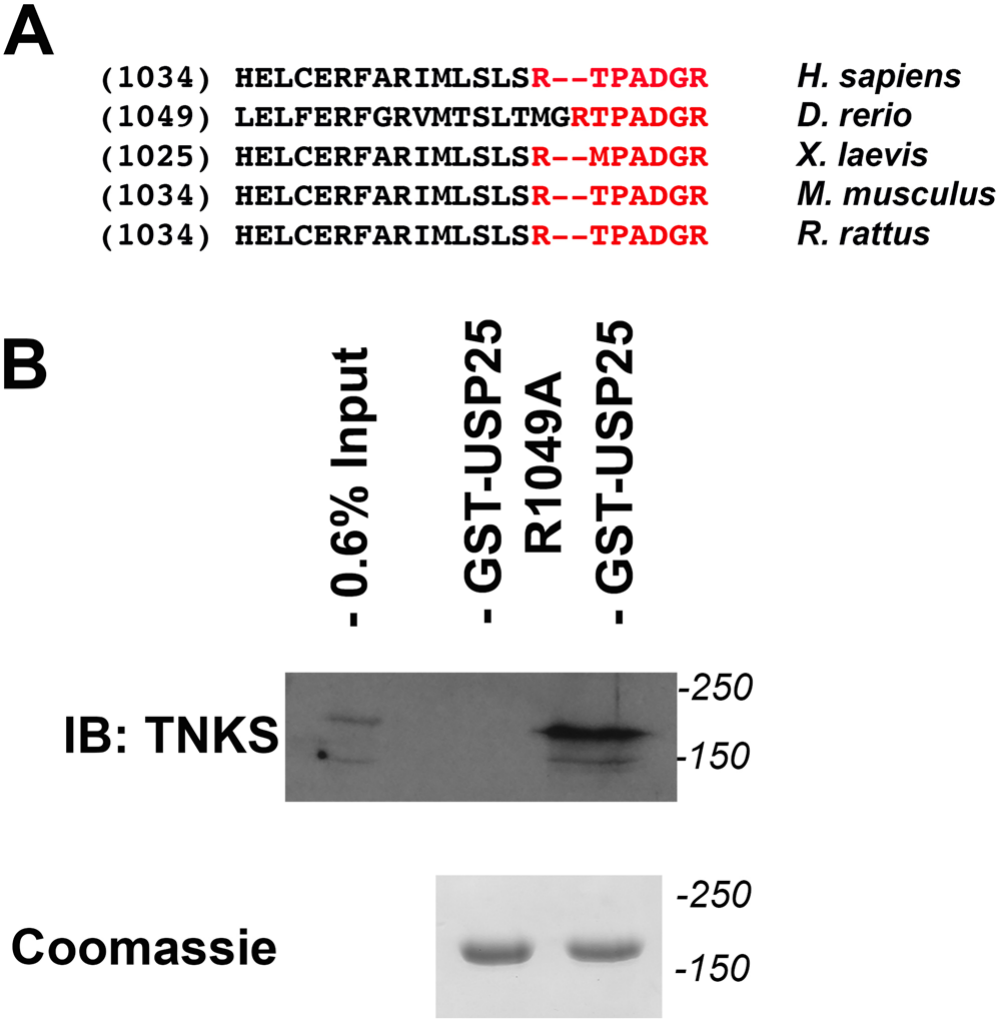
Mutation of the conserved arginine in the TNKS binding motif of USP25 abrogates binding from adipocyte cell lysate. (**A**) The C-termini of known sequences for human and mouse USP25, and predicted sequences for rat, frog (*Xenopus Laevis*) and zebrafish (*Danio rerio*) from the UniProt database were aligned using Vector NTI (ThermoFisher). The conserved tankyrase binding motif is in red and bold. Figures in brackets correspond to the first amino acid residue shown in the sequence alignment. (**B**) The ability of GST-USP25 and a derivative harbouring a mutation in the conserved TNKS binding motif (GST-USP25-R1049A) to bind TNKS from 3T3-L1 adipocytes was assessed by incubating 10 μg of each GST fusion immobilised on Sepharose beads with lysate prepared from 2, 10 cm dishes of 3T3-L1 adipocyte cell lysate (4 mg total protein in 400 μl). Immunoblot analysis was used to detect TNKS in the pulldown (upper panel) and in 1.5% of the input lysate and a Coomassie stained gel of the fusion proteins used for the bindings (lower panel). Full-length blots/gels are presented in the Supplementary Information.

Our hypothesis postulates that TNKS serves to recruit USP25 to GSVs. USP25 harbours a conserved TNKS binding motif (RXXPDG) (Fig. 1A) and has been identified as a TNKS-interacting protein in HEK293 cells^14,15^. To characterize interaction between these two proteins in insulin-sensitive cells, we expressed and purified recombinant GST-USP25 from bacteria, and carried out pulldown assays from adipocyte lysates. Figure 1B shows that GST-USP25 precipitated endogenous TNKS from a 3T3-L1 adipocyte lysate. Mutation of the conserved arginine in the TNKS binding motif of USP25 (R1049A) abrogates this binding, demonstrating that the RXXPDG motif in USP25 facilitates interaction of the deubiquitinase from adipocytes with TNKs (Fig. 1B).

### TNKS and its binding partner USP25 are enriched on GLUT4-containing membranes but do not translocate to the plasma membrane in response to insulin

**Figure 2:**
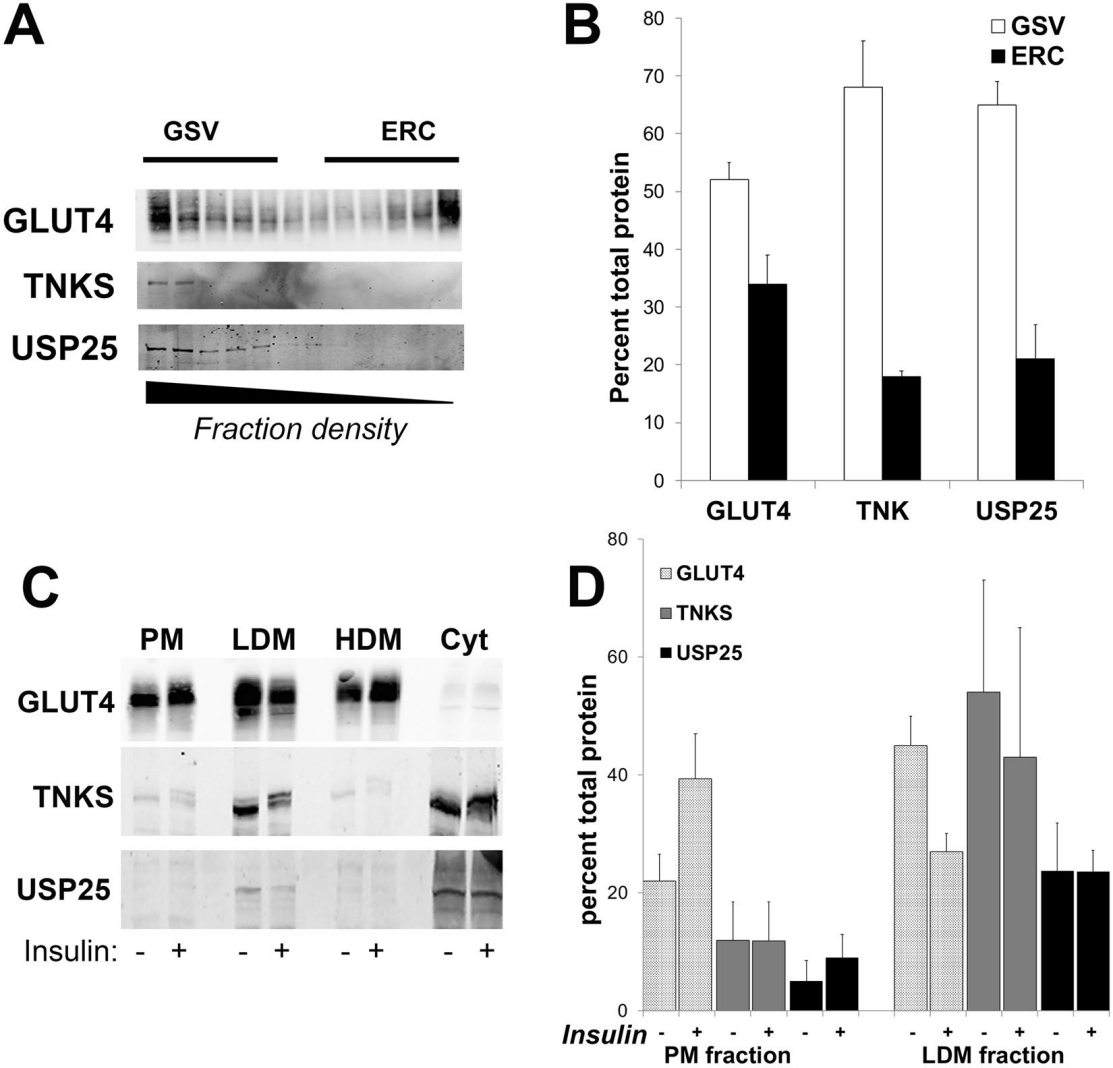
TNKS and USP25 cofractionate with GLUT4 in low-density microsome (LDM) fractions of adipocytes but do not translocate to the plasma membrane in response to insulin. (**A**) Low density microsomes (LDMs) prepared from serum starved 3T3-L1 adipocytes were further fractionated in a self-forming iodixanol gradient. Twelve fractions, collected from the bottom of the gradient were subject to immunoblot analysis to determine the distribution of GLUT4, TNKS and USP25. Note that fraction 1 is not a pellet, but the most dense fraction of the gradient. (**B**) ImageJ software was used to quantify the proportion of GLUT4, TNKS and USP25 in the two separate pools of GLUT4 contained within LDMs, fractions 1–5 and 8–12, previously shown to be enriched in GSVs and endosomal recycling compartments (ERC) respectively^16^. Error bars represent ± SEM, from three separate biological replicates of the fractionation. (**C**) Serum starved 3T3-L1 adipocytes were incubated with (+) or without (−) 100 nM insulin for 30 minutes before being fractionated into soluble (Sol) and plasma membrane (PM), LDM and high density microsome (HDM)–enriched fractions. Immunoblot analysis was used to detect GLUT4, TNKS and USP25 in each fraction. (**D**) ImageJ software was used to quantify the proportion of total GLUT4, TNKS and USP25 in the PM and LDM fractions shown in (**C**). Error bars represent ± SEM, from three experiments of this type. Full-length blots/gels are presented in the Supplementary Information.

Sorting of GLUT4 into insulin-responsive GSVs requires TNKS^11^. TNKS is found in the perinuclear region of adipocytes where it colocalises with GLUT4^13^. Iodixanol gradient centrifugation of adipocyte membranes resolves GLUT4-containing membranes into two distinct populations with the denser of the two containing GSVs^16^. Figure 2(A,B) shows that TNKS is highly enriched in the more dense GLUT4-containing fractions consistent with its role in sorting into GSVs^11^. USP25 is also found in these fractions supporting our hypothesis that interaction with TNKS serves to position this deubiquitinase to function in GLUT4 sorting (Fig. 2A,B).

GSV-resident proteins such as GLUT4 and IRAP translocate to the plasma membrane in response to insulin from internal membranes, contained within low density microsomes (LDMs) obtained via differential centrifugation of adipocytes^1^. In contrast to GLUT4, the amount of TNKS and USP25 did not decrease in the LDM nor increase in plasma membrane fractions exhibit significant increases in response to insulin-stimulation (Fig. 2C,D).

### USP25 is required to maintain GLUT4 stability and insulin-responsiveness

**Figure 3:**
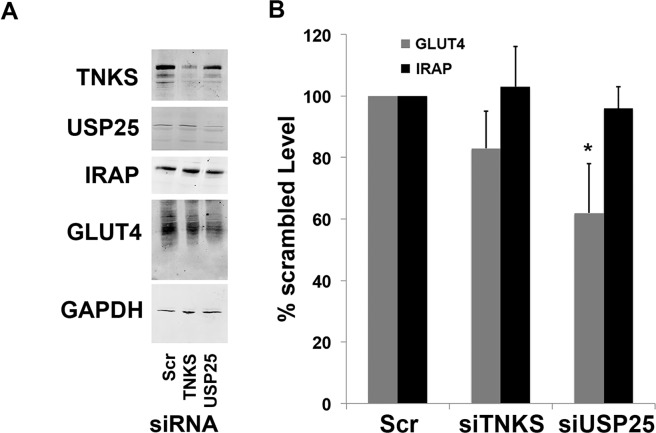
siRNA mediated depletion of USP25 in adipocytes results in reduced levels of GLUT4 protein. (**A**) 3T3-L1 adipocytes were treated with 50 nM siRNA against either TNKS or USP25, or a scrambled control siRNA (Scr) using Mirus TransIT TKO reagent on days 6 and 7 post-differentiation before being harvested on day 8 and subject to immunoblot analysis for the indicated proteins. (**B**) ImageJ software was used to quantify levels of GLUT4 and IRAP, normalised against GAPDH, in cells treated with siRNA against TNKS or USP25, expressed as a percentage of their levels in cells treated with the scrambled control siRNA. Error bars represent ± SEM, n = 3; asterisk denotes significance. Full-length blots/gels are presented in the Supplementary Information.

Data presented in Fig. 1 demonstrate that USP25 from adipocytes can bind TNKS, and Fig. 2 shows that while both these proteins are present in GSV-enriched subcellular fractions of adipocytes neither translocate to the plasma membrane in response to insulin. To test the hypothesis that USP25 is positioned to rescue GLUT4 from lysosomal degradation we investigated the effect of USP25 depletion on GLUT4 levels. siRNA mediated knockdown of USP25 in 3T3-L1 adipocytes resulted in a decrease in the levels of GLUT4 compared to control cells (Fig. 3). Consistent with our previous study^11^, we also found that TNKS knockdown in 3T3-L1 adipocytes did not affect GLUT4 levels (Fig. 3).

**Figure 4:**
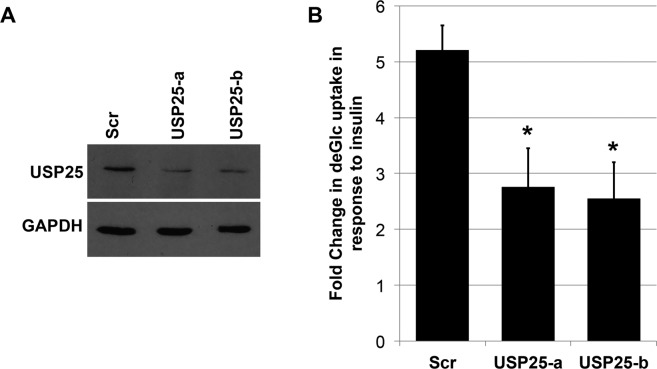
siRNA mediated depletion of USP25 from adipocytes decreases insulin-stimulated glucose uptake. 3T3-L1 adipocytes were treated with 50 nM of one of two siRNAs against USP25 (-a and -b; N.B. USP25-a is the siRNA shown in Fig. 3A), or a scrambled control siRNA (Scr) using Mirus TransIT TKO reagent on days 6 and 7 post-differentiation before being analysed on day 8. (**A**) Immunoblot analysis was performed to confirm knockdown of USP25 using GADPH as a loading control. (**B**) The ability of insulin (100 nM, 30 min) to stimulate [^3^H]-2-deoxyglucose uptake into these cells was assayed (each point is the mean of triplicate determinations, ±SEM, expressed as fold increase over basal transport rate). Full-length blots/gels are presented in the Supplementary Information.

To further investigate the role of USP25 in GLUT4 sorting, we examined the consequences of USP25 depletion on the ability of 3T3-L1 adipocytes to respond to insulin. Adipocytes treated with control siRNA exhibited ~5-fold increase in the rate of glucose uptake in response to insulin as measured by uptake of radiolabeled 2-deoxyglucose uptake assay (Fig. 4). Knockdown of USP25 reduced this insulin response by ~50% consistent with a similar decrease in levels of GLUT4 (Fig. 3).

## Discussion

Our previous finding that ubiquitination of GLUT4 in 3T3-L1 adipocytes is essential for sorting of the transporter into insulin-responsive GSVs raised the question as to how this modification differs from ubiquitination that targets integral membrane proteins to the MVB pathway^5,7^.

Our finding that only ~0.1% of cellular GLUT4 in adipocytes is ubiquitinated at steady state led us to suggest that GLUT4 ubiquitination is a transient modification that is reversed once its role of directing the transporter into GSVs has been fulfilled^5^. We identified USP25 as a candidate DUB to regulate this process through its interaction with IRAP, via TNKS^13,14^. This is an attractive model given that IRAP and TNKS are required for the formation of GSVs and insulin-regulated trafficking of GLUT4, but GLUT4 is not required for insulin-regulated trafficking of IRAP^10,11^. Further support for this novel hypothesis comes from our work using the yeast *Saccharomyces cervisiae* as a model system to study GLUT4 trafficking^5,17^. While expression of human GLUT4 in yeast results in its ubiquitination as in mammalian cells, in yeast this modification results in vacuolar degradation of the transporter^5,17^. These data suggest that while some aspects of regulated trafficking through the endosomal system are conserved through evolution, including ubiquitination and GGA-dependent sorting, yeast lack some of the molecular mechanisms that exist in insulin-responsive cells to localise GLUT4 to insulin-responsive GSVs^5,17^. One possibility is that while the molecular machinery to sort GLUT4 into the endosomal system is present in yeast the link with a deubiquitinating enzyme evolved later, explaining why yeast degrade GLUT4 rather than store it intracellularly^5,17^. Furthermore, a constitutively ubiquitinated version of GLUT4, constructed by fusing ubiquitin in-frame to the C-terminus, is rapidly degraded in both yeast and insulin-sensitive cells; this is consistent with our model as this fusion is unlikely to be a DUB substrate and thus the protein is internalized into MVBs and subsequently degraded.

In this study we have tested further aspects of our model that sorting of GLUT4 into GSVs requires the DUB USP25 to be positioned by interaction with TNKS. We have established that USP25 from adipocytes can form a complex with TNKS, as predicted by yeast-2-hybrid analysis and interactions between these two proteins in non insulin-sensitive cells^14,15^. These data support our hypothesis that TNKS links the GSV component IRAP to the DUB USP25. Previous work has shown that depletion of either IRAP or TNKS1 from 3T3-L1 adipocytes blunts insulin responsive glucose uptake^11^. Additionally, IRAP knockout mice, despite exhibiting normal glucose tolerance, have a reduced insulin-responsive glucose uptake and GLUT4 protein levels in isolated tissues^12^. These findings are compatible with our model of an IRAP-TNKS1-USP25 complex regulating GLUT4 traffic.

To test the hypothesis that USP25 is positioned to rescue GLUT4 from lysosomal degradation we investigated the consequences on GLUT4 levels of USP25 depletion. siRNA-mediated knockdown of USP25 in 3T3-L1 adipocytes resulted in a decrease in both cellular GLUT4 levels (Fig. 3) and insulin-stimulated glucose uptake (Fig. 4). Although USP25 knockdown may exert these effects either by increasing GLUT4 degradation or reducing de novo biosynthesis of GLUT4, we consider the former the most likely explanation based upon known functions of ubiquitination. Future experiments, including investigations of whether inhibition of lysosomal hydrolases causes accumulation of ubiquitinated GLUT4 in cells depleted of USP25 will address this issue, but these experiments are confounded by our finding that robust knockdown of USP25 requires ongoing rounds of transfection with siRNA; a process that requires endosomal acidification. Such future studies are likely to reveal any role a ubiquitination/deubiquitination cycle plays in regulating total cellular levels of GLUT4, a long-lived protein whose steady state levels are regulated through lysosomal degradation^18,19^. DUB activity has been shown to be required for recycling of several plasma membrane proteins, including the epithelial sodium channel^20^, the Drosophila Wnt co-receptor Frizzled^21^ and the beta-2 adrenergic receptor^22^. Our findings however, extend this paradigm, and are novel in terms of sorting of a protein from the TGN through the endosomal system.

Consistent with previous studies^11,23^, we found that TNKS knockdown in 3T3-L1 adipocytes did not reduce total GLUT4 levels in adipocytes, which might have been anticipated if this protein is required to position USP25 to deubiquitinate GLUT4 to rescue it from lysosomal degradation. TNKS knockdown in these cells does, however, result in attenuated insulin-stimulated delivery of GLUT4 to the plasma membrane and glucose transport, as well as altered GLUT4 localisation within the endosomal system under basal conditions^11,23^, and TNKS knockout mice show an expanded pool of cellular GLUT4^24^. Effects of TNKS knockdown in 3T3-L1 adipocytes are reproduced using pharmacological inhibition of TNKS which concomitantly alters association of TNKS with axin and the motor protein KIF3A^11,23^. These data raise the possibility that TNKS has a dual role in GLUT4 trafficking; utilizing both its scaffolding and enzymatic properties. One possibility is that the levels of TNKS knockdown achieved is adipocytes is not sufficient to mislocalise all USP25, and that remaining is sufficient to deubiquitinate GLUT4 and rescue it from lysosomal degradation. It is important to note that in L6 myotubes, TNKS knockdown, or treatment of the cells with pharmacological inhibitors of TNKS activity, not only impairs insulin-stimulated glucose uptake but also reduces cellular levels of several GSV proteins including GLUT4^25^, perhaps indicating different physiological roles of fat and muscle.

Intriguingly, TNKS PARsylation is known to direct ubiquitination of protein substrates including axin via the E3 ligase RNF146^26^. This finding can be used to extend our model so that IRAP recruitment of TNKS facilitates TNKS PARsylation of GSV components, including GLUT4, tagging them for ubiquitination to facilitate sorting into GSVs. USP25, recruited through interaction with TNKS, could then deubiquitinate GLUT4, trapping it in GSVs and preventing its trafficking to the lysosome.

Precisely where in the endosomal system such a cycle of ubiquitination/deubiquitination of GLUT4 occurs requires, and warrants, further investigation. GGA proteins are most associated with sorting of proteins from the TGN into the endosomal system through recognition of attached ubiquitin moieties^6,7^, suggesting that GLUT4 may become ubiquitinated as it travels through the TGN. GLUT4 becomes trapped in a ubiquitin-enriched late endosomal compartment upon overexpression of dominant-negative ESCRT mutants and is unable to respond to insulin^27^; suggesting that the ubiquitinated form of the transporter transits through the late endosomal compartment where the ubiquitin-dependent ESCRT sorting machinery functions prior to entering GSVs. These two observations are consistent with our model as the compartments that make up the endosomal system are highly dynamic in nature. Consistent with our finding that, unlike GSV-resident proteins, USP25 and TNKS do not translocate to the plasma membrane in response to insulin, neither USP25 nor TNKS were identified in a comprehensive proteomic analysis of GSVs^28^, suggesting that these proteins function before GLUT4 enters its final storage depot in insulin-sensitive cells.

## Methods

### Cell culture

3T3-L1 fibroblasts (American Tissue Culture Collection) were grown and differentiated into adipocytes as described^29^. Prior to experimental analyses, cells were incubated in serum-free media for 2 hours and incubated with or without insulin as detailed in figure legends. 2-deoxy-D-glucose (deGlc) transport was assessed as previously described^30^. 3T3-L1 adipocytes were treated with 50 nM siRNA against either TNKS or USP25, or a scrambled control siRNA (Scr) using Mirus TransIT TKO reagent on days 6 and 7 post-differentiation before being harvested on day 8 and subject to immunoblot analysis for the indicated proteins.

### GST-fusion pull-downs

GST-USP25^31^ and a version with Arg_104_ mutated to Ala were purified from bacteria and used to pull down proteins from an adipocyte lysate as described^5^.

### Subcellular fractionation

Fractionation of adipocytes into plasma membrane (PM), high and low-density membranes (HDM and LDM respectively) and cytosolic (cyt) fractions was performed using a well-characterised and widely used protocol^32^. LDM fractions were further separated using Iodixanol gradient centrifugation as described^16^. To quantify the proportion of GLUT4 within GSV-enriched fractions, immunoblot signals from lanes 1–5 were quantified, combined and expressed as a fraction of the total GLUT4 signal across all fractions. Immunoblot analysis was performed using antibodies against GLUT4 and USP25 that have been described elsewhere^33,34^. Polyclonal antibodies for IRAP were from Paul Pilch (Boston University School of Medicine). Other antibodies were purchased from Ambion (anti-GAPDH mouse monoclonal; clone 6C5) and Santa Cruz biotechnology (anti-TNKS rabbit polyclonal). Immunoblot signals were quantified using ImageJ (National Institutes of Health, Bethesda, MD) software.

## Supplementary information

Supplementary information
