# Supplementary material for: The deubiquitinating enzyme USP25 binds tankyrase and regulates trafficking of the facilitative glucose transporter GLUT4 in adipocytes
Source: Sci Rep. 2019 Mar 18;9:4710. doi: 10.1038/s41598-019-40596-5 (PMC6423145; doi:10.1038/s41598-019-40596-5)

Supplementary information for:

The deubiquitinating enzyme USP25 binds tankyrase and regulates trafficking of the facilitative glucose transporter GLUT4 in adipocytes

Jessica B.A. Sadler[2], Christopher A. Lamb[2], Cassie R. Welburn[2], Iain S. Adamson[2], Dimitrios Kioumourtzoglou[1], Nai-Wen Chi[3], Gwyn W. Gould[2*] and Nia J. Bryant[2*]

[1]Department of Biology, University of York, York YO10 4HJ U.K.

[2]Henry Wellcome Laboratory of Cell Biology, Institute of Molecular Cell and Systems Biology, College of Medical Veterinary and Life Sciences, University of Glasgow, Glasgow G12 8QQ U.K.

[3]Department of Medicine, University of California, San Diego, La Jolla, CA 92093 U.S.A.

[*]nia.bryant@york.ac.uk
[*]gwyn.gould@glasgow.ac.uk

Blots for Figure 1.

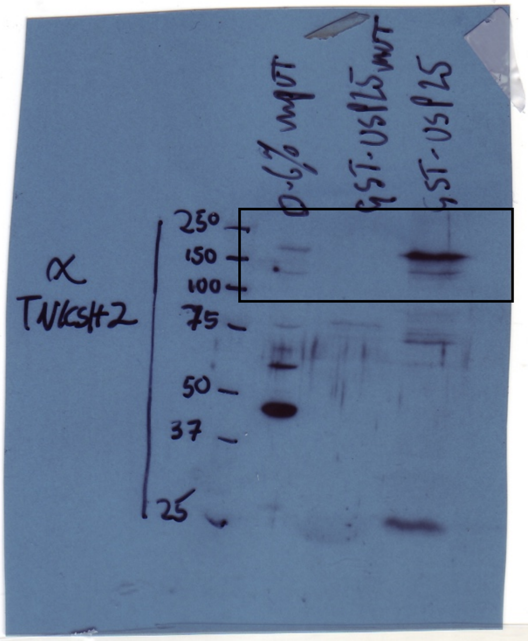

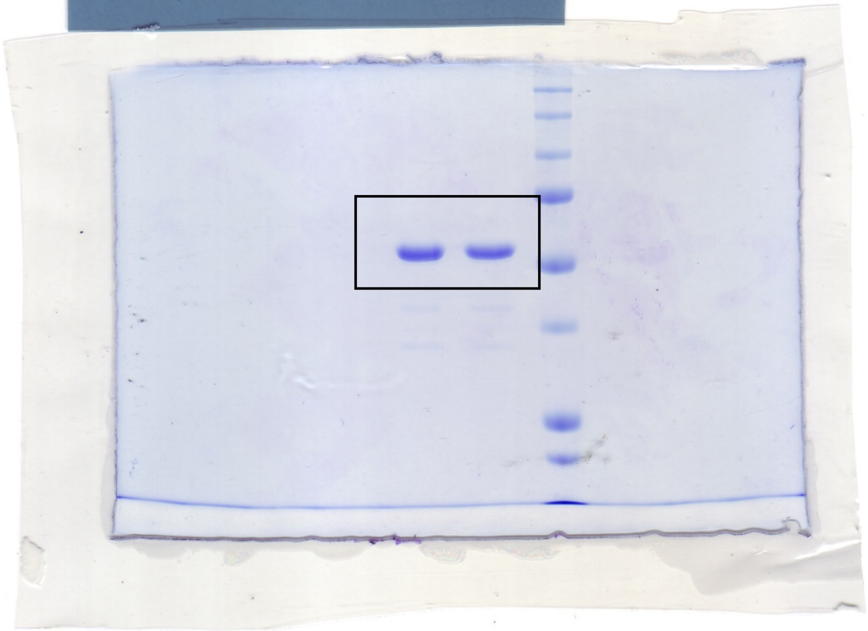

Blots for Figure 2A.

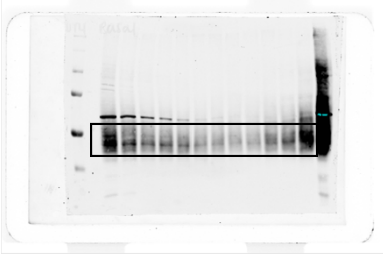

GLUT4 blot, positive control on extreme right not shown in figure.

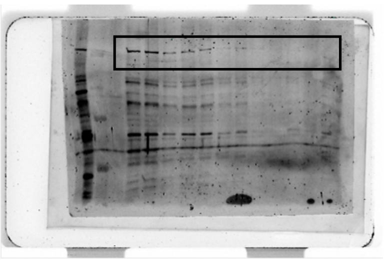

USP25 blot, positive control left lane, followed by marker, empty lane then data in figure.

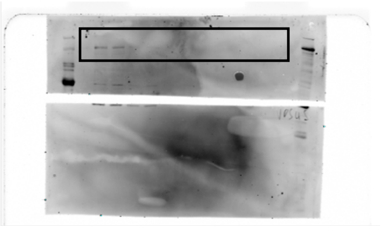

TNKs blot. Positive control at right. Markers in left lane then samples shown on figure. Lower panel a different antibody and data not used in this manuscript.

Blots for Figure 2C.

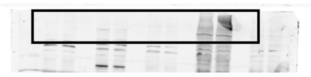

USP25 blot, extreme two right hand lanes not for this experiment.

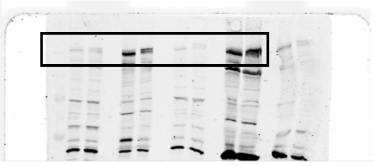

TNKs blot, extreme two right hand lanes not for this experiment

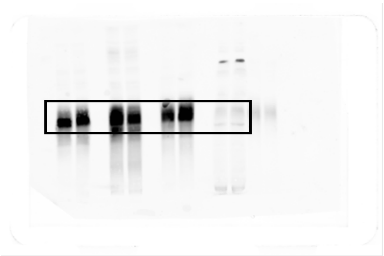

GLUT4 blot, extreme two lanes not for this experiment

<u>Blots for Figure 3.</u>

TNKS

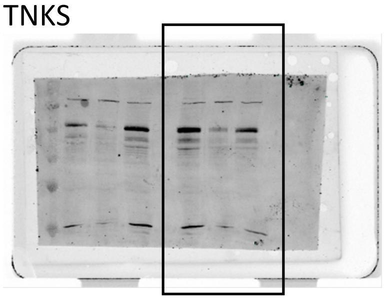

USP25

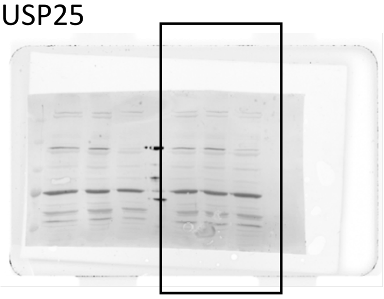

IRAP

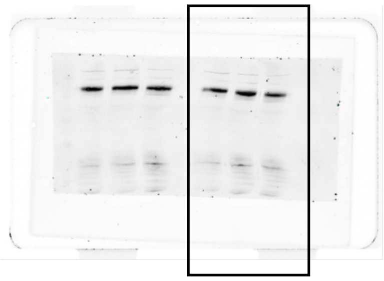

GLUT4

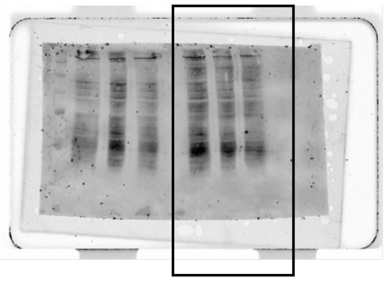

GAPDH

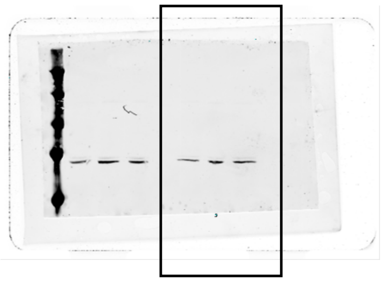

Blots for figure 4.

Upper is anti-USP25

Lower is anti-GAPDH

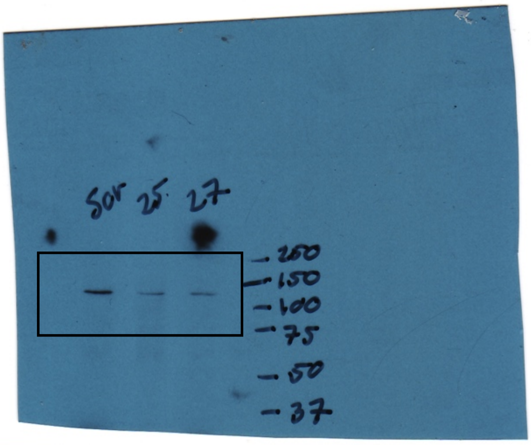

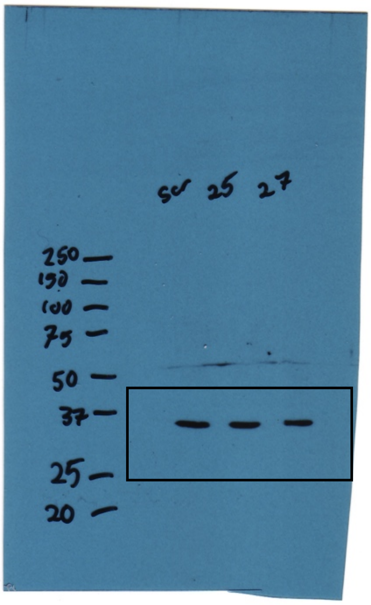

Supplement: Supplementary file 1 — Supplementary information [file 41598_2019_40596_MOESM1_ESM.pdf]
